# A discourse network analysis of UK newspaper coverage of the “sugar tax” debate before and after the announcement of the Soft Drinks Industry Levy

**DOI:** 10.1186/s12889-019-6799-9

**Published:** 2019-05-02

**Authors:** Christina H. Buckton, Gillian Fergie, Philip Leifeld, Shona Hilton

**Affiliations:** 1 MRC/CSO Social and Public Health Sciences Unit, University of Glasgow, 200 Renfield Street, Glasgow, G2 3AX UK; 2 Department of Government, University of Essex, Colchester Campus, Colchester, CO4 3SQ UK; 3 School of Social and Political Sciences, University of Glasgow, University Avenue, Glasgow, G12 8QQ UK

**Keywords:** Public health policy, UK soft drinks industry levy, SDIL, SSB tax, Discourse network analysis, Media content analysis, Unhealthy commodity industries

## Abstract

**Background:**

On 6th April 2018, the UK Government introduced the Soft Drinks Industry Levy (SDIL) as a mechanism designed to address increasing prevalence of obesity and associated ill health by reducing sugar consumption. Given that the successful introduction of upstream food and nutrition policies is a highly political enterprise involving multiple interested parties, understanding the complex network of stakeholders seeking to influence such policy decisions is imperative.

**Methods:**

Media content analysis was used to build a dataset of relevant newspaper articles, which were analysed to identify stakeholder agreement or disagreement with defined concept statements. We used discourse network analysis to produce visual representations of the network of stakeholders and coalitions evident in the debate as it was presented in UK newspapers, in the lead up to and following the announcement of the Soft Drinks Industry Levy in the UK, from May 2015 to November 2016.

**Results:**

Coding identified 3883 statements made by 214 individuals from 176 organisations, relating to 47 concepts. Network visualisations revealed a complex network of stakeholders with clear sceptical and supportive coalitions. Industry stakeholders appeared less united in the network than anticipated, particularly before the SDIL announcement. Some key industry actors appeared in the supportive coalition, possibly due to the use of corporate social responsibility rhetoric. Jamie Oliver appeared as a dominant stakeholder, firmly embedded with public health advocates.

**Conclusion:**

This study highlights the complexity of the network of stakeholders involved in the public debate on food policies such as sugar tax and the SDIL. Polarisation of stakeholders arose from differences in ideology, focus on a specific policy and statements about the weight of evidence. Vocal celebrity policy entrepreneurs may be instrumental in gaining public and policy makers’ support for future upstream regulation to promote population health, to facilitate alignment around a clear ideology.

**Electronic supplementary material:**

The online version of this article (10.1186/s12889-019-6799-9) contains supplementary material, which is available to authorized users.

## Background

On 6th April 2018, the United Kingdom (UK) Government introduced a Soft Drinks Industry Levy (SDIL) commonly referred to as the “sugar tax”. The levy was intended to reduce sugar consumption, primarily through reformulation by soft drinks manufacturers to reduce sugar content and avoid paying the levy [1, 2]. Excess consumption of free sugars is a contributory factor in the rising prevalence of non-communicable diseases (NCDs), both as a direct cause [3, 4] and through the contribution to energy imbalance resulting in obesity [5]. In 2015 the World Health Organisation (WHO) issued guidance on sugars consumption for adults and children, recommending reducing intake of free sugars to less than 10%, and ideally less than 5%, of total energy intake [6]. In the lead up to UK Government’s introduction of the SDIL, there was much policy debate about how to achieve this goal [7, 8], with evidence suggesting the need for upstream policy intervention [9–11].

Products containing tobacco and alcohol have long been subjected to taxation, which was traditionally seen as a way of raising revenue for public spending [12]. More recently, there is increased emphasis on taxation of these products to promote population health, given the inverse relationship between price and consumption [13–16]. However, the role of price increases through taxation in food and nutrition policy is not clear-cut [12, 17, 18]. Sassi et al. note that, in the case of foods, the value of using taxes depends upon their design and the context in which they are applied [12]. Specifically, if people are aware that a product is taxed for public health reasons, rather than to raise revenue, they may be more likely to change their consumption [18]. A number of governments around the world have adopted taxation of selected energy-dense foods and drinks [19]. A recent policy analysis describing 13 international case studies concluded that taxation seemed to have the desired effects on prices and consumption of energy-dense products [19].

The successful introduction of upstream food and nutrition policies is a highly political enterprise with multiple vested interests [20]. Stakeholders include politicians, the commercial sector, public health professionals, academics, non-government organisations, government advisors, journalists, public figures such as celebrities, and grassroots organisations [21]. The example of the Danish ‘fat tax’, implemented in 2011 and abolished after only 15 months despite recent evidence of its success [22], highlights tensions between stakeholders. Bødker et al. note that active industry lobbying and judicial actions undermined policy support [23]. This illustrates the highly politicised nature of introducing new policies when the evidence base is limited and policies are opposed by those with vested corporate interests [20]. Research suggests that one reason governments do not pursue upstream approaches to food and nutrition policy is the power and influence of the food industry [20, 24–26]. Understanding industry influence in such public policy debates is challenging given the complex network of stakeholders involved. As Smith et al. note, understanding this network of stakeholders, their relationships, and interactions is necessary for elucidating advocacy strategies to counter industry and protect public health [27].

One theory of policy change or stability, the Advocacy Coalition Framework, suggests that policy subsystems are formed around competing advocacy coalitions, which are based on shared ideological belief systems. Changes in core policy preferences by political actors result in changes in the balance of coalitions and hence policy shifts [28, 29]. Leifeld suggests that the articulation of policy beliefs by interest groups in the media and other arenas, in the form of discourses, reveals their policy preferences and encourages other stakeholders in the policy debate to support them or reveal their opposition [30]. Mapping of such agreement or disagreement in discursive forums can produce representations of advocacy coalitions and policy networks [30]. Thus, network analysis can be used to explore the complex interactions and alliances that stakeholders form in attempting to influence government policy [21, 31–33].

Social network analysis has been used in tobacco control policy debates to highlight the polarisation of opposing coalitions and draw attention to the complex processes of consensus-seeking, alliance-building, and strategic action, which are integral to the development of policy [33]. In the case of Minimum Unit Pricing (MUP) for alcohol, discourse network analysis (DNA) [34, 35], a combination of category-based content analysis and social network analysis, has been used to provide insights into the formation of discourse coalitions and cast light on the complexity of alignments between stakeholders engaged in the MUP debate as cited in UK newspapers [36]. Whilst Hilton et al. found that both proponents and opponents of the SDIL actively engaged with the news media to promote framings that would advance their interests [37], there is little evidence on how the complex network of coalitions and alliances of stakeholders formed and changed during the SDIL policy debate, and how those networks may have impacted the Conservative Party’s shift in policy position and adoption of the regulation.

This study uses DNA to offer the first visual representation of the network of stakeholders and advocacy coalitions apparent in UK newspaper content in the lead-up to, and following, the announcement of the SDIL in the UK. Specifically we aim to: (i) determine the membership of coalitions active in the debate and how these developed over the period of policy debate (May 2015 to November 2016); (ii) explore alliances and cleavages across different sectors/interest groups; and (iii) generate network insights on industry lobbying, campaigning, and policy advocacy.

## Methods

### Data extraction and content analysis

We employed media content analysis methods established by Hilton and colleagues to build a dataset of relevant newspaper articles [38–40]. We selected eight UK and three Scottish newspapers with their Sunday counterparts. Further detail is provided in Additional file 1. The publications with high circulation figures were chosen to represent three genres of newspaper (quality/broadsheet, mid-market, and tabloid) covering a range of readerships profiles in relation to age, social class, and political alignment [41].

A 19-month period from May 2015 to November 2016 was selected to cover key events and publications surrounding the SDIL policy debate. Specifically: (i) the publication of research on health harms of excessive sugar consumption and evidence for appropriate policy action [6, 42, 43]; (ii) the House of Commons sugary drinks tax policy debate [7]; (iii) the publication of an early evaluation of a sugar tax policy in Mexico [44]; (iv) the announcement of the SDIL in March 2016 [45]; and (v) the public and industry consultation on SDIL proposals [46, 47] (Table 1).

**Table 1:** Timeline of events leading to the introduction of the UK Soft Drinks Industry Levy

Date	Event/publication
Jun-Sep 2014	Public consultation on the Scientific Advisory Committee on Nutrition (SACN) draft report “Carbohydrates and Health”
Mar 2015	World Health Organisation guideline “Sugars intake for adults and children”
Jul 2015	Scientific Advisory Committee on Nutrition report “Carbohydrates and health”
Sep 2015	Jamie Oliver parliamentary petition “Introduce a tax on sugary drinks in the UK to improve our children’s health” – received 155,516 signatures
Oct 2015	Public Health England report “Sugar reduction: The evidence for action”
Nov 2015	Parliamentary debate on public petition “Introduce a tax on sugary drinks in the UK to improve our children’s health”
Jan 2016	BMJ study “Beverage purchases from stores in Mexico under the excise tax on sugar sweetened beverages: observational study”
Mar 2016	Budget announcement: proposals for a Soft Drinks Industry Levy (SDIL)
June 2016	Referendum on UK’s membership of the European Union
Aug-Oct 2016	Public consultation on SDIL proposals
Aug 2016	UK Government obesity strategy “Childhood obesity: A plan for action”
Mar 2017	Budget announcement: confirmation of plans for a UK-wide SDIL
Mar 2017	Health Affairs study “Sustained consumer response: evidence from two-years after implementing the sugar sweetened beverage tax in Mexico”
Apr 2018	Introduction of the SDIL in the UK

After testing various terms, the search terms [“sugar” OR “beverage”] (in the headline) AND [“tax” OR “levy”] (anywhere in the text) were used to identify relevant articles in the Nexis database [48]. The search identified 995 articles, 834 after removal of duplicates. All articles were read to determine whether they met the pre-defined inclusion criteria, i.e.: (i) “sugar tax”/SDIL being the primary focus; (ii) citing one or more stakeholders (as a direct quotation or a comment that was directly attributable to the stakeholder); and (iii) the type of article being news, commentary or feature piece. After exclusions, 511 articles were included for analysis.

All 511 articles were exported to the software tool Discourse Network Analyzer (DNA) [35, 49, 50]. Using the DNA software, researchers coded extracts of newspaper text which featured stakeholders’ arguments on “sugar tax”, SSB tax, or the SDIL as “statements”. Statements are ensembles of four variables: individual stakeholder’s name (where available), organisational affiliation of the stakeholder (the “actor”), the argument referred to by the stakeholder (the “concept”), and a dichotomous variable for the stakeholder’s agreement or disagreement with the concept (“agreement”). Two researchers independently double-coded a 10% sample of articles using an initial set of concepts based on earlier analysis of the minimum unit pricing (MUP) for alcohol debate [36]. After discussing inconsistencies and making refinements to the concept statements and coding framework to include new concepts specific to the SDIL debate, all articles were coded. In total, coding identified 3883 statements made by 214 individuals from 176 organisations, relating to 47 concepts. Details of the stakeholders and concepts coded are provided in Additional file 2.

### Network visualisation and analysis

A weighted stakeholder × stakeholder matrix was created using the DNA software, where common agreement or disagreement between stakeholders on individual concepts was represented by ties and their relative weights. The “subtract” transformation with “average activity normalisation” [49] was applied. The subtract transformation measures argumentative similarity in excess of differences of opinion. That is, a tie weight between two actors is expressed as the number of concepts on which these actors have identical opinions minus the number of concepts on which these actors have diverging opinions. The normalisation of tie weights ensures that only argumentative similarity, but not the rate at which stakeholders issue statements, is considered for the calculation of tie weights. This is done by dividing the tie weights by the average number of concepts the two actors mention throughout the policy debate. A threshold value of ≥0.4 was applied to the tie weights in the resulting network, and tie weights lower than this threshold value were replaced by 0. This was done to retain only relatively robust argumentative similarity as ties in the network [49], in order break down the complexity of the debate to a manageable level and make coalitions visible.

The stakeholder × stakeholder network was imported into the network visualisation software Visone [51] to map-out visually the stakeholders and their coalitions. Exported network matrices are provided in additional data (Additional files 3, 4 and 5). Girvan-Newman edge-betweenness community detection, a common graph clustering algorithm [52], was applied to the network in order to identify coalitions as cohesive subgroups with similar argumentative patterns. The coalitions were highlighted in the network visualisation as blue hyperplanes. Stakeholder types were highlighted using colours, and the frequency of citations for each stakeholder was visualised as the size of the respective node. Network measures were used to describe the overall network and main clusters: size (number of nodes), centralisation (a measure of how skewed the distribution of all actors’ connections is [53]), density (number of ties as a proportion of the theoretical maximum [54]), and external ratio (number of ties to nodes outside the identified cluster as a proportion of total ties).

In addition to the overall network visualisation, separate network visualisations were analysed for two time-periods: May 2015 – mid-January 2016 and mid-January 2016 – November 2016. This allowed the examination of the formation of coalitions and any changes in position for stakeholders in the debate both before and after the shift in government policy on SSB taxation, which became apparent in newspaper reporting of the debate in January 2016 [55].

An analysis of bipolarisation over time was used to illustrate the degree of polarisation into two distinct coalitions over the time-period considered. Polarisation is the tendency of the factions, or clusters, in the discourse network to be segregated and not show any overlap in policy beliefs. For example, if the supportive cluster and the sceptical cluster show very little similarity in arguments and positions, the two coalitions can be said to be relatively polarised. In contrast, if there are many intermediaries who blur the boundaries between the coalitions, the extent of bipolarisation is relatively small. Bipolarisation can change over time, and we analysed how polarised the coalitions were at any point in the observation period using a temporally smoothed curve of network modularity. Modularity is a common measure in network analysis for measuring the tendency of a network to have clearly delineated clusters [56]. More specifically, the following measures were applied to the discourse network to measure bipolarisation.

At any time point, bipolarisation was computed by first applying 11 different graph clustering techniques that permit specifying the number of desired clusters k = 2 in advance; then computing network modularity [56] for each of the 11 cluster solutions; and finally choosing the maximal modularity value among the 11 values as a measure of bipolarisation. Bipolarisation through modularity of the optimal k = 2 cluster structure expresses the tendency of the network to fall into exactly two clusters, or “coalitions.” This bipolarisation measure was smoothed over time by executing these steps for a window of 200 statements, moving the time window forward by one statement, and re-computing the bipolarisation measure each time until the end of the empirical policy debate was reached. The bipolarisation values for each consecutive 200-statement window were visualised in a time series diagram, with each time window centred around the respective date, and a LOESS (Local Polynomial Regression) smoother was fitted through the resulting curve to indicate trends more clearly.

## Results

### Network overview – supporters and sceptics’ coalitions

**Fig. 1:**
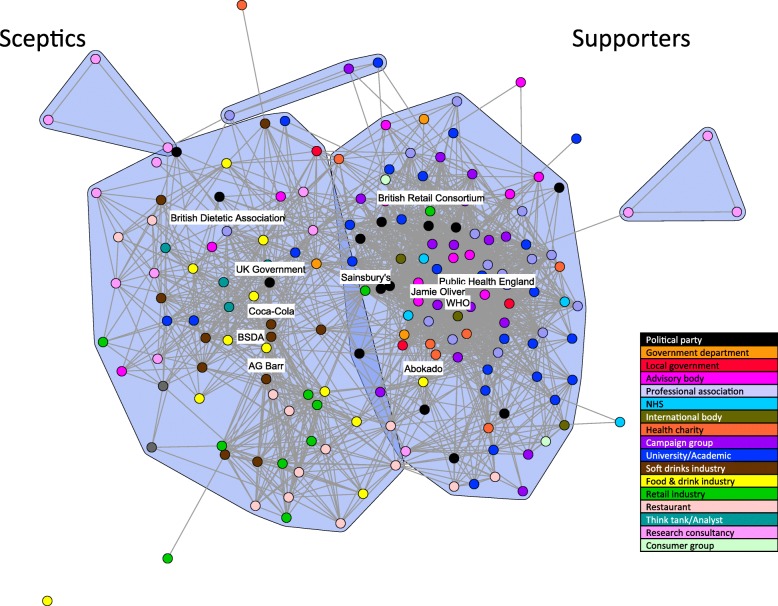
Discourse network for all stakeholders in the full time period. Legend: Nodes are uniform size to ensure the high number of stakeholder nodes are visible (BSDA: British Soft Drink Association; WHO: World Health Organisation)

The coalitions active in the sugar debate in UK media coverage are shown in Fig. 1. The two main coalitions can be characterised as either broadly supportive or sceptical of fiscal policies to control sugar consumption as a way of dealing with obesity (henceforth referred to as “supportive” and “sceptical” coalitions respectively). There are more stakeholders in the supportive coalition (*n* = 95) than in the sceptical coalition (*n* = 65) (Table 2). The supportive coalition consists primarily of organisations categorised as health charities, health campaign groups, professional associations, advisory bodies, NHS representatives, and international bodies such as the World Health Organisation (WHO). Prominent, frequently cited supporters include Public Health England, the World Health Organisation and Jamie Oliver (an English chef and restaurateur, more recently known as a campaigner for healthy food for children). In contrast, the sceptical coalition comprised representatives from the food and drink industry, specifically the soft drinks industry, retailers, restaurants and economic analysts and think tanks. Prominent sceptics in this coalition include the British Soft Drinks Association, Coca-Cola, AG Barr, and the UK Government.

**Table 2:** Network measures for the two main coalitions

Network measure	Full network	Supportive cluster	Sceptical cluster
Size (number of nodes)	176	95	65
Centralisation	29.8%	39.7%	36.9%
Density	0.13	0.29	0.24
Total external ratio		0.05	0.12

A number of stakeholder types do not appear exclusively in one coalition or the other. Political parties, government departments, commercial research organisations, and academics are spread across both coalitions. This spread reflects the complexity of the debate in relation to policy responses to obesity and views on the likely effectiveness of taxation changing over time. A few stakeholders appear isolated in the opposite coalition to other stakeholders of the same type. For example, the British Dietetic Association is the only professional association to appear in the sceptical coalition, and only three representatives from the food and drink industry (Abokado, Sainsbury’s and the British Retail Consortium) appear in the supportive coalition (Fig. 1).

The total external ratio (Table 2) is defined as the number of extra-coalition ties of an actor over all ties the actor possesses, averaged over all actors in a given coalition. It provides a measure of the number of co-agreements and co-disagreements between stakeholders in one coalition with stakeholders in the opposing coalition. The comparison of the two coalitions indicates a lower total external ratio for the supportive coalition. This suggests the stakeholders in this coalition were less likely to agree with arguments made by stakeholders in the sceptical coalition. The higher external ratio for the sceptical coalition may be due to the common agreement that obesity is a significant health problem requiring attention and reflects social responsibility messages used by the food and drink industry in adopting similar framings to those of public health advocates. In line with the external ratio, the supportive coalition also has a slightly greater degree of centralisation (Table 2). Centralisation measures the extent to which a few selected actors dominate a coalition by having more links to others than the remaining actors.

### Development of coalitions over time

**Fig. 2:**
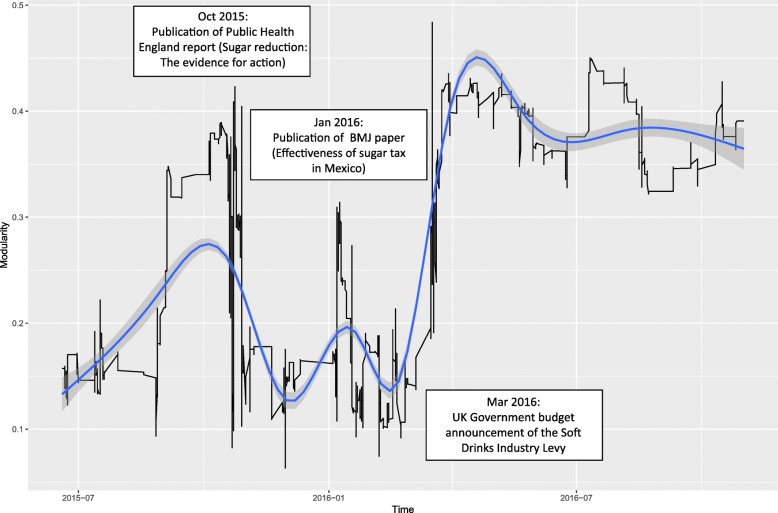
Network bipolarisation from July 2015 to November 2016

Temporal analysis of bipolarisation modularity measures indicates that the tendency of the network to fall into precisely two coalitions changed in line with key policy events and publications (Fig. 2). Peaks in the bipolarisation curve over time show how the discourse network developed more clear-cut, mutually opposing coalitions in the policy process over time. The higher the curve, the stronger the tendency of the coalitions to distance themselves from the respective other coalition, the fewer brokers exist between coalitions, and the more homogeneity of arguments within each coalition. The first peak coincides with the publication of the Public Health England report “Sugar reduction: the evidence for action” in October 2015 [43] and a period of intense campaigning by Jamie Oliver, culminating in the parliamentary debate in November. The smaller second peak occurs in January 2016 around the time of the publication of a BMJ report suggesting that the tax on sugar-sweetened beverages in Mexico was associated with reductions in purchases of taxed beverages and increases in purchases of untaxed beverages [44]. The final sharp rise follows the government’s change in policy position in January and the announcement of the SDIL in March 2016. The elevated level of bipolarisation continued during the SDIL consultation period.

**Fig. 3:**
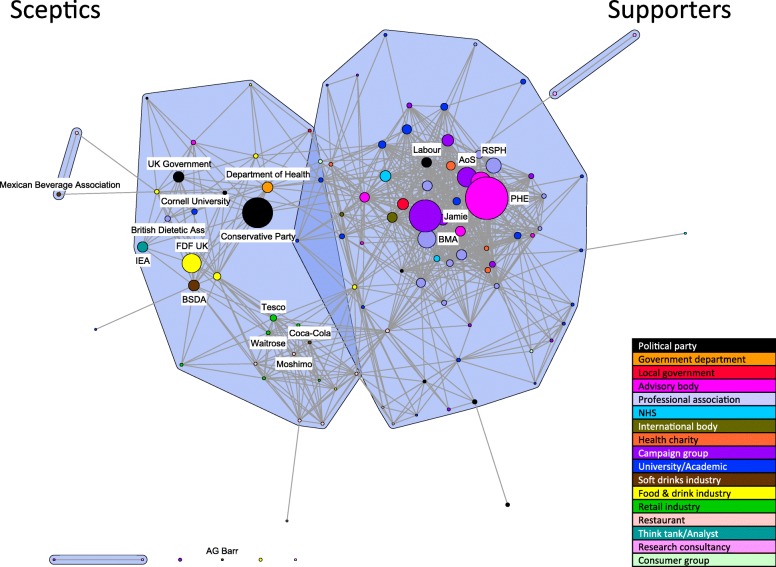
Discourse network highlighting the position and relative prominence of key stakeholders: pre Jan 2016. Legend: Selected stakeholders are labelled to highlight the position and relative prominence of key actors. (AoS: Action on Sugar; BMA: British Medical Association; BSDA: British Soft Drinks Association; FDF UK: UK Food and Drink Federation; IEA: Institute of Economic Affairs; Jamie: Jamie Oliver; PHE: Public Health England; RSPH: Royal Society for Public Health)

**Fig. 4:**
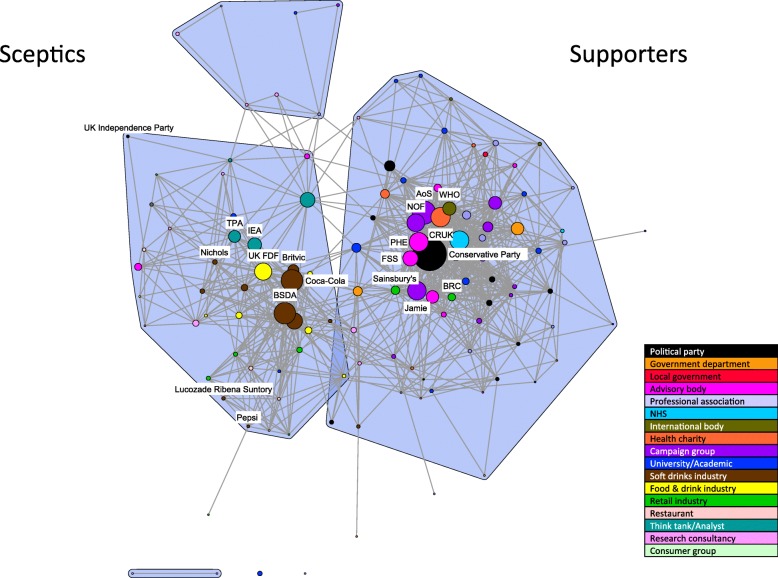
Discourse network highlighting the position and relative prominence of key stakeholders: post Jan 2016. Legend: Selected stakeholders are labelled to highlight the position and relative prominence of key actors. (AoS: Action on Sugar; BRC: British Retail Consortium; BSDA: British Soft Drinks Association; CRUK: Cancer Research UK; UK FDF: UK Food and Drink Federation; FSS: Food Standards Scotland UK; IEA: Institute of Economic Affairs; Jamie: Jamie Oliver; NOF: National Obesity Forum; PHE: Public Health England; TPA: TaxPayer’s Alliance; WHO: World Health Organisation)

We present network visualisations for two time-periods, before and after mid-January 2016 (Figs. 3 and 4). This coincided with the government’s apparent shift in policy position and the publication of evidence of the efficacy of a tax on sugar-sweetened beverages (SSBs) in Mexico in the British Medical Journal on 6 January 2016 [44]. Network measures show a 40% increase in sceptics entering the debate after Jan 2016, particularly from the soft drinks industry, together with higher density and centralisation for the sceptical coalitions both before and after January 2016 (Table 3). This is possibly because the specific policy option mentioned at this time was a tax on sugary drinks.

**Table 3:** Network measures for the two main coalitions: pre and post Jan 2016

Network measure	Supportive cluster		Sceptical cluster	
	Pre Jan 2016	Post Jan 2016	Pre Jan 2016	Post Jan 2016
Size (number of nodes)	67	68	29	40
Centralisation	36.2%	34.0%	43.1%	41.1%
Density	0.28	0.26	0.38	0.32
Total external ratio	0.03	0.04	0.13	0.10

#### Pre January 2016: emphasis on defining the problem and perceptions of a general sugar tax

The network during the early time-period reflects the period of intense campaigning by sugar taxation advocates, including Jamie Oliver, campaign group Action on Sugar, advisory body Public Health England, and professional associations, most notably the British Medical Association and the Royal Society for Public Health (Fig. 3). The node representing Jamie Oliver is the second largest in the network after Public Health England, indicating the scale of his presence in the debate in this period, larger than any of the soft drinks manufacturers or political stakeholder nodes. The node is entrenched within the coalition broadly supportive of action, with no ties to industry representatives. At the time Jamie Oliver appeared to embark on personal crusade: highlighting the problems arising from excess sugar consumption, particularly for young people, in the press and his TV documentary “Sugar Rush”; calling for an SSB tax as a solution to this problem, going as far implementing such a tax in his own restaurants and initiating a parliamentary petition on the subject; and demanding personal action from the then Prime Minister David Cameron [57]. Campaign groups, professional bodies and health charities have an average external ratio of zero at this time, indicating close agreement and no ties to any stakeholders in the sceptics’ coalition (Table 4). Unsurprisingly the Labour party appears in the supportive coalition in opposition to the stance of the Conservative party in power at the time.

**Table 4:** Average external ratios by type of stakeholder organisation: pre and post Jan 2016

Stakeholder organisation type	Average external ratios	
	Pre Jan 2016	Post Jan 2016
Political party	0.18	0.05
Government department	0.31	0.07
Local government	0.21	0.00
Advisory body	0.13	0.07
Professional association	0.00	0.14
NHS	0.05	0.00
International body	0.06	0.00
Health charity	0.00	0.19
Campaign group	0.00	0.01
University/Academic	0.10	0.12
Soft drinks industry	0.18	0.11
Food and drink industry	0.13	0.15
Retailer/Retail association	0.10	0.15
Restaurant	0.23	0.00
Think tank/Analyst	0.00	0.14
Research consultancy	0.13	0.13
Consumer group	0.06	–

By comparison, nodes representing the food and drink industry are smaller, indicating less activity, and they are spread across two apparent sub-clusters within the sceptical coalition (Fig. 3). One sub-cluster comprises retail organisations (most prominently Tesco’s and Waitrose) restaurants (for example Moshimo) and two food and drink manufacturers (most prominently Coca-Cola). The other comprises a more diverse mix of stakeholders including politicians, government departments and advisors (most significantly the Conservative Party, UK Government and Department of Health), representatives of food and drink manufacturers such as the UK Food and Drink Federation and the British Soft Drinks Association, one think tank (the Institute for Economic Affairs), one academic (Cornell University) and one professional association (the British Dietetic Association). Stakeholders in sub-cluster one appear unified around concepts such as “the food and drink industry is already taking voluntary action”, and “industry plays an active role in public health promotion”, while those in sub-cluster two share concepts emphasising the inappropriate nature of taxation (as an intervention in the market and a regressive tax) and that “working in partnership with industry is a better way of tackling obesity”.

Only four representatives of soft drinks manufacturers appear at this time, the British Soft Drinks Association, Coca-Cola, AG Barr, and the Mexican Beverage Association. Overall, the soft drinks industry stakeholder group has an average external ratio of 0.18 (Table 4), suggesting a relatively high number of links to the supportive coalition. Statements made by industry representatives in newspapers suggest their use of corporate social responsibility rhetoric to soften anti-legislation messages. It is possible that this strategy increases their agreement with supporters and hence the relatively high external ratio for this stakeholder category.

The UK Government and the Conservative party are broadly aligned with the manufacturers and opposing stakeholders in their statements during this period and appear in the opposite coalition to government advisory bodies such as Public Health England. Similarly, the Department of Health is firmly embedded in the sceptical coalition with ties to the Conservative party and food and drinks industry spokespeople, specifically the UK Food and Drink Federation and the British Soft Drinks Association.

#### Post January 2016: alliances and cleavages following policy announcement

The network illustrating the later time-period shows apparent movement of key stakeholders (Fig. 4). There is an increase in activity by food and drink industry representatives as indicated by the size and number of brown (soft drinks manufacturers and trade associations) and yellow (food and drink industry more generally) nodes, perhaps reflecting the newly established policy focus on sugary drinks. The British Soft Drinks Association, Coca Cola, and the UK Food and Drink Federation are particularly prominent and central to the sceptics’ coalition, suggesting their leadership in framing arguments against the SDIL. Representation from soft drinks manufacturers increases from four to eleven, with Britvic, Lucozade Ribena Suntory, Nichols, and Pepsi among the new stakeholders appearing in the sceptical coalition. However, there continues to be a relatively high average external ratio for stakeholders in this group (0.15 for the food and drink industry generally and 0.11 for the soft drinks industry specifically) (Table 4). These findings suggest some level of agreement between sceptics in the food and drink industry and supporters of the policy and once again this seems to be related to ongoing corporate social responsibility messages.

The nodes in the sceptical coalition appear to have converged, from two sub-clusters into one. Key stakeholders are brought together by concepts criticising SSB taxation as “an unfairly punitive tax on the soft drinks industry” and “questioning its likely effectiveness as a policy measure”; perhaps reflecting the emergence of the SDIL as the favoured policy option. The number of think tanks and analysts in the debate increases from one to four, all appearing in the sceptical coalition. Two in particular align with industry stakeholders, the Institute for Economic Affairs and The Taxpayers Alliance, with external ratios of zero.

While the soft drinks industry appears to align around arguments against the SDIL, retail organisations are less consistent in their opposition; two key stakeholders, Sainsbury’s and the British Retail Consortium, appear in the supportive coalition (Fig. 4), and the average external ratio for retail stakeholders is 0.15 (Table 4). Such industry cleavages may reflect the degree to which the specific industry stakeholders consider themselves directly threatened by the policy; as the retail sector is less likely to be damaged by SDIL, they can distance themselves from anti-legislation messages and reinforce their role in promoting public health.

Across the time-period, the UK Conservative party shifts from a position aligned with industry to a position at the core of the supportive coalition, closely aligned with Public Health England and Food Standards Scotland. The timing of this shift may be partly explained by contextual factors (Table 1). Namely: the publication of persuasive evidence of health harms related to excess sugar consumption, particularly for young people [43]; the subsequent period of intense campaigning by Jamie Oliver with attendant media coverage and the evidence emerging from Mexico on the potential effectiveness of SSB taxation [44]. In this second time-period, all political parties move into the supportive coalition, with one exception (the UK Independence Party), and the overall external ratio for the political party stakeholder group reduces from 0.18 to 0.05 (Table 4). This suggests greater alignment with health campaigners and other policy supporters (most prominently Jamie Oliver, the National Obesity Forum, Action on Sugar, Cancer Research UK and the World Health Organisation) and may have been facilitated by the policy focus on the SDIL rather than more general discourse on sugar taxation.

## Discussion

This study highlights the complexity of the network of stakeholders and their involvement in the debate on sugar tax and the SDIL. Our analysis identified the involvement of a large number of stakeholders, and some apparent divisions within the food and drink industry and commonalities between some industry segments and public health advocates. The coalitions changed over time, with peaks in bipolarisation coinciding with publication of evidence on the health harms of excess sugar consumption and policy announcements. In the first time-period, polarisation appears to arise primarily from ideological positioning (whether or not taxation of any kind is an appropriate measure), whereas later it comes from contradictory positions on whether or not SSBs are the appropriate target of policy regulation. The impact of the unexpected policy announcement in early 2016 may have contributed to the increased bipolarisation of the network and alignment within both supportive and sceptical coalitions, as stakeholders united to strengthen their positions in response to a specific policy.

Corporate social responsibility strategies represent an important mechanism by which controversial, or potentially socially harmful, industries seek to mitigate the level of controversy arising from their business activities [58]. Fooks et al. suggest that corporate social responsibility activities allow corporate stakeholders, such as those in the tobacco industry, to justify ethically problematic market actions that promote economic interests over public health concerns [59]. One example observed in the alcohol industry is the dissemination of health information to the public while misrepresenting the evidence of health harms associated with their products [60, 61]. In the case of the SSB industry, manufacturers have sought to emphasise the importance of physical activity over calorie restriction in dealing with obesity; as exemplified by Coca-Cola’s investment in the Global Energy Balance Network [62]. Our analysis of discourse networks suggests a more complex interplay between protecting profitability and corporate social responsibility strategies in the case of the SDIL debate. Different sectors of the food and drink industry present different views, resulting in unanticipated commonality between some industry sectors and public health campaigners, and cleavages between industry segments. Retailers and retail associations, manufacturers and restaurants were not entirely aligned in their media statements. Parts of the retail sector were situated outside the sceptical coalition, including Sainsbury’s, the British Retail Consortium, and Abokado (a retailer and manufacturer with a mission to “lead happier and healthier lives”), reinforcing their position as being “part of the solution” [63]. In contrast, soft drinks manufacturers appeared in the sceptical coalition alongside think tanks and economic analysts, drawn together by similar statements characterising the policy as unfair, an inappropriate intervention in the market and too simplistic. This set of arguments is familiar from alcohol industry opposition to the Minimum Unit Pricing policy [64], and other so-called unhealthy commodity industries [65].

The prominence of public health advocates and campaigner nodes in the supportive coalition perhaps reflects intense lobbying by these stakeholders prior to the announcement of the policy. A recent review of public health advocacy to reduce health inequalities revealed inconsistencies in framing of policy and a lack of coherence between theory and action, which resulted in multiple barriers to consistent public health advocacy [66]. In contrast, the networks revealed by this study of the public debate on the SDIL suggest unity among public health advocates on the scale of the problem and the importance of regulatory action. Two factors that may have facilitated alliances in support of the SDIL may have been that, firstly, the levy was designed to both encourage industry reformulation and reduce individual consumption, and secondly, the levy targeted a commodity that can be linked directly to health harms with no nutritional benefit.

Conversely, we suggest that the food and drink industry is inconsistent in their lobbying tactics. In studies of other industries’ efforts to influence policy, stakeholders are portrayed as employing consistently effective tactics to oppose upstream regulation, including the use of a playbook of succinct, well-drilled messages delivered by central spokespeople [61, 65, 67, 68]. In contrast, this study demonstrates that the structure of the sceptical coalition pre January 2016 takes the form of two sub-coalitions representing industry sub-sectors; one emphasising public health framing of the debate (supermarkets and retailers), and the other focusing on ideological arguments (food and drink industry, politicians and think tanks). Post January 2016, the sceptical coalition appears to be more aligned. It is dominated by soft drinks manufacturers, their representatives and think tanks, with Coca-Cola and the British Soft Drinks Association at its heart. Representatives of retailers and restaurants either become peripheral to the coalition or move to the supportive coalition, along with most politicians. Stakeholders in this more cohesive sceptical coalition are united by concepts criticising SSB taxation as a regressive, unfair, punitive tax on the soft drinks industry and questioning its likely effectiveness as a policy measure, while reinforcing corporate social responsibility rhetoric. This suggests that soft drinks manufacturers were less coordinated before the SDIL announcement, perhaps believing that existing voluntary agreements with government, in the form of the Public Health Responsibility Deal [69], would protect them from further regulation. In other words, the industry may have been caught off-guard. This suggestion is supported by the position of the UK Government and the Conservative party in the network in the early time-period, when their statements were aligned with industry representatives in the sceptics’ coalition.

Kingdon defines policy entrepreneurs as *“people who are willing to invest their resources in pushing their pet proposals or problems”* [70]. As such they can be instrumental in setting the policy agenda, highlighting solutions to problems, getting the attention of policy makers and thus facilitating policy change [71]. More recently Pepin-Neff and Caporale have highlighted the importance of high-profile individuals in bringing about political change [72]. The size and central position of Jamie Oliver’s node in the supportive coalition is suggestive of his role as such a celebrity policy entrepreneur in the public debate on sugar tax and highlights the increasing sophistication and importance of public health activists. Sustain, an alliance of organisations advocating for food and agriculture policies and practices that enhance health and welfare, published an analysis entitled “How the sugary drinks tax was won: 10 lessons for committed campaigners” [73]. They also highlight the importance of working with high-profile advocates to take public health campaigns to a new level of recognition and impact, as well as facilitating alliances of medical and public health professionals, academics, journalists and politicians [73]. Our findings support this recommendation, showing Jamie Oliver occupying a dominant central position at the heart of the supportive coalition, particularly in the period before the SDIL policy announcement.

Although our use of DNA provides a number of novel insights into the network of stakeholders active in the sugar debate, the study has some limitations. The data was limited to the statements attributed to stakeholders in UK print media. Newspaper debates are only one arena among several in which political discourse unfolds, and this research cannot comment on the parliamentary or judicial arenas, or any discussions that occur behind closed doors. However, understanding public debates in the media arena offers a useful “door opener to the backstage of politics”, as Wodak and Meyer argue [74]. Additionally, restricting data to quotes or comments cited in newspapers that were directly attributable to stakeholders minimised any editorial impact. The application of a strength of tie threshold value of ≥0.4 removed a number of inter-stakeholder associations and allowed for a focus on only the most robust ties in the network. A final limitation arose from restricting the research to print media, which may have excluded stakeholders who operate exclusively in social media. For example, the campaign group People Against Sugar Tax did not appear in this analysis. However, the researchers’ shared experience of media research suggests the number of such organisations is small.

## Conclusion

In conclusion, polarisation of stakeholders visible in the media debate on SDIL arose from differences in ideology, focus on a specific policy and statements on the weight of evidence. The food and drink industry stakeholders appeared to be caught off-guard by the specific SDIL policy announcement and seemed less cohesive as a coalition than might have been anticipated based on research on other manufacturers of unhealthy commodities. Formation of advocacy coalitions in support of upstream regulation seems dependent on alignment around a clear ideology and policy objectives. A vocal celebrity policy entrepreneur could provide an important locus for this, in the way that Jamie Oliver appeared to do in this example of sugar tax and the SDIL.

Use of DNA methods offers the first visual map of the SDIL network and allows exploration of a complex set of relationships involved in framing public health policies. Further research is needed to explore what motivated the complex dynamic in this contested policy debate, potentially through interviews with the stakeholders involved. Further DNA analyses may be conducted around broader public health problems, not focussed on a specific policy measure, but instead using a health problem as a starting point and analysing actors’ convergence on a set a policies over time to further our understandings of health policy making.

## Additional files

Additional file 1: Appendix A Publications included in the sample. List of newspaper titles selected for inclusion in the process to build a dataset of relevant articles. (DOC 47 kb)

Additional file 2: Appendix B Detail of stakeholders and concept statements coded. Data consists of two tables. Table 1 provides details of the stakeholders coded as either agreeing or disagreeing with one or more concept statements cited in the debate on the Soft Drinks Industry Levy, ie: Type of Organisation (colour indicates the colour used to highlight the organisation type in the network diagrams), stakeholder organisation and abbreviation used in the network diagrams. Table 2 describes the concept statements. (DOC 105 kb)

Additional file 3: Network matrix data for the full time period May 2015-Nov 2016. Network data exported from DNA software used in analysis for Figs. 1 and 2. (CSV 285 kb)

Additional file 4: Network matrix data for the time period prior to Jan 2016. Network data exported from DNA software used in analysis for Fig. 3. (CSV 113 kb)

Additional file 5: Network matrix data for the time period post Jan 2016. Network data exported from DNA software used in analysis for Fig. 4. (CSV 141 kb)

## Abbreviations

ANPRAC: Mexican Beverage Association
BSDA: British Soft Drinks Association
CSR: Corporate social responsibility
DNA: Discourse Network Analyzer
EDF: Energy dense food
HMT: Her Majesty’s Treasury
LOESS: Local Polynomial Regression
LRS: Lucozade Ribena Suntory
MUP: Minimum unit pricing
NCD: Non-communicable disease
NHS: National Health Service
PHE: Public Health England
SACN: Scientific Advisory Committee on Nutrition
SDIL: Soft Drinks Industry Levy
SSB: Sugar-sweetened beverages
UCI: Unhealthy commodity industry
WHO: World Health Organisation
