# Supplementary material for: A discourse network analysis of UK newspaper coverage of the “sugar tax” debate before and after the announcement of the Soft Drinks Industry Levy
Source: BMC Public Health. 2019 May 2;19:490. doi: 10.1186/s12889-019-6799-9 (PMC6498658; doi:10.1186/s12889-019-6799-9)
Supplement: Supplementary file 1 — Appendix A Publications included in the sample. List of newspaper titles selected for inclusion in the process to build a dataset of relevant articles. (DOC 47 kb) [file 12889_2019_6799_MOESM1_ESM.doc]

**Appendix A: List of publications included in the sample**

1. The Guardian / The Observer

2. Independent

3. Times / Sunday Times

4. Daily Telegraph / Sunday Telegraph

5. Daily Mail / Mail on Sunday

6. Express / Sunday Express

7. Sun / News of the World

8. Mirror / Sunday Mirror

9. Daily Record / Sunday Mail

10. Scotsman / Scotland on Sunday

11. Herald / Sunday Herald
