# Supplementary material for: A discourse network analysis of UK newspaper coverage of the “sugar tax” debate before and after the announcement of the Soft Drinks Industry Levy
Source: BMC Public Health. 2019 May 2;19:490. doi: 10.1186/s12889-019-6799-9 (PMC6498658; doi:10.1186/s12889-019-6799-9)
Supplement: Supplementary file 2 — Appendix B Detail of stakeholders and concept statements coded. Data consists of two tables. Table 1 provides details of the stakeholders coded as either agreeing or disagreeing with one or more concept statements cited in the debate on the Soft Drinks Industry Levy, ie: Type of Organisation (colour indicates the colour used to highlight the organisation type in the network diagrams), stakeholder organisation and abbreviation used in the network diagrams. Table 2 describes the concept statements. (DOC 105 kb) [file 12889_2019_6799_MOESM2_ESM.doc]

**Appendix B: Detail of stakeholders and concept statements coded**

**Table 1: Stakeholders coded as agreeing or disagreeing with one or more concept statements**

| Type of Organisation^(1)^ | | Stakeholder organisation | Abbreviation^(2)^ |
| --- | --- | --- | --- |
| Political party |  | - Australian Government - Australian Greens - Conservative - Labour - Liberal Democrat - Mexican politicians - National Action Party (PAN) - Plaid Cymru - Scottish Conservatives - Scottish Labour - Scottish Liberal Democrats - Scottish National Party - UK Government - UK Independence Party | - Aust Gov - Aust Greens - Conservative - Labour - Lib Dems - Mex politicians - PAN - Plaid Cyrmu - Sc Con - Sc Lan - Sc Lib Dems - SNP - UK Gov - UKIP |
| Government department |  | - Dep for Environment, Food & Rural Affairs - UK Government - DoH - UK Government - Treasury | - DEFRA - DoH - HMT |
| Local government |  | - Brighton & Hove Council - Liverpool City Council - Wolverhampton Council | - B&H CC - Liverpool CC - Wolv CC |
| Advisory body |  | - Behavioural Insights Team - Chief Medical Officer - Commons Health Select Committee - Food Responsibility Network (PRD) - Food Standards Scotland - Local Government Association - Office for Budget Responsibility - Public Health England (EA) - Scientific Advisory Committee on Nutrition - Scotland's Chief Medical Officer - Scottish Food Commission - Scottish Health Survey - Scottish Parliament Information Centre | - BIT - CMO - CHSC - FRN - FSS - LGA - OBR - PHE - SACN - Sc CMO - SFC - SHS - SPIC |
| Professional association |  | - Academy of Medical Royal Colleges - Association of Directors of Public Health - British Dental Association - British Dietetic Association - British Medical Association - Faculty of Public Health - General Practitioners - Royal College of Dental Surgeons - Royal College of General Practitioners - Royal College of Midwives - Royal College of Paediatrics & Child Health - Royal College of Physicians - Royal College of Physicians Edinburgh - Royal College of Psychiatrists - Royal College of Surgeons Ireland - Royal Society for Public Health | - AMRC - ADPH - BDA - BDieteticA - BMA - FPH - GPs - RCDS - RCGP - RCM - RCP&CH - RCP - RCPE - PCPsy - RCS(Ire) - RSPH |
| NHS |  | - East Lancashire NHS Trust - NHS Confederation - NHS England - NHS Greater Glasgow and Clyde | - EL NHSTrust - NHS Conf - NHS England - NHS GGC |
| International body |  | - National Institute of Pharmacy and Nutrition (Budapest) - Organisation for Economic Co-operation and Development - World Health Organisation | - NIPN Hungary - OECD - WHO |
| Health charity |  | - British Heart Foundation - Cancer Research UK - Child Growth Foundation - Diabetes UK - European Society of Lifestyle Medicine - Heart Research UK - Irish Heart Foundation - World Cancer Research Fund | - BHF - CRUK - CGF - Diabetes UK - ESLM - HRUK - IHF - WCRF |
| Campaign group |  | - Action on Sugar - Alianza por la Salud Alimentaria   (Nutritional Health Alliance)   - Ceres - Children's Food Campaign - Jamie Oliver - National Obesity Forum - Nourish Scotland - Obesity Action Scotland - Obesity Health Alliance - Obesity Stakeholder Group - Soil Association - Sustain - The Richmond Group - UK Active - UK Health Forum - World Obesity Federation | - AoS - ASA - Ceres - CFC - Jamie - NOF - NS - OAS - OHA - OSG - Soil Ass - Sustain - TRG - UKActive - UKHF - WOF |
| University/Academic |  | - British Medical Journal - Brighton University - Centre for Dementia Prevention   (Edinburgh University)   - Centre for Diet and Activity Research (Cambridge University) - City University London - Cornell University - Duke University - Harvard School of Public Health - Karolinska Institute - King's College London - London Metropolitan University - National Institute of Public Health Mexico (INSP) - Queen Mary University - St Andrews University - Stirling University - Tufts University - University College London - University of Birmingham - University of California - University of Cambridge - University of Dundee - University of Glasgow - University of Liverpool - University of North Carolina - University of Oxford - University of Reading | - BMJ - Brighton Uni - CDP - CEDAR - CUL - Cornell Uni - Duke Uni - Harvard SPH - Karolinska Inst - KCL - LMU - NIPH Mexico - QMU - St Andrew’s - Stirling Uni - Tufts Uni - UCL - U Birmingham - U California - U Cambridge - U Dundee - U Glasgow - U Liverpool - U N Carolina - U Oxford - U Reading |
| Soft drinks industry |  | - AG Barr - Australian Beverages Council - British Soft Drinks Association - Britvic - Coca-Cola - Irish Beverage Council - Lucozade Ribena Suntory - Mexican beverage Association - Nichols - Pepsi - Sibberi - Wild Orchid | - AG Barr - ABC - BSDA - Britvic - Coca-Cola - IBC - LRS - ANPRAC - Nichols - Pepsi - Sibberi - Wild Orchid |
| Food and drink industry |  | - AB Sugar - Abokado - Associated British Foods - British Sugar (part of AB Foods) - Cadbury - Food and Drink Federation - Food and Drink Industry Ireland - Kellogg's - Mars Food - Scotland Food and Drink - Sugar Nutrition UK - UK Sugar Bureau - Unilever | - AB Sugar - Abokado - ABF - BS - Cadbury - FDF - FDI Ireland - Kellogg’s - Mars - F&D Scotland - SNUK - UKSB - Unilever |
| Retailer/Retail association |  | - Aldi - Asda - British Retail Consortium - Lidl - Marks & Spencer - Sainsbury's - Scottish Grocers Federation - Scottish Retail Consortium - Tesco - Waitrose | - Aldi - Asda - BRC - Lidl - M&S - Sainsbury’s - SGF - SRC - Tesco - Waitrose |
| Restaurant |  | - British Beer & Pub Association - Burger Brothers - Costa - Frankie & Benny's - Jamie Oliver Restaurant Group - Leon - McDonalds - Moshimo - Pizza Hut - Starbucks - Tortilla - Union Jacks - Wetherspoons | - BBPA - Burger Bros - Costa - F&Bs - JO Rest Grp - Leon - MsDonalds - Moshimo - Pizza Hut - Starbucks - Tortilla - Union Jacks - Wetherspoons |
| Think tank/Analyst |  | - Adam Smith Institute - Institute for Fiscal Studies - Institute of Economic Affairs - Taxpayer's Alliance | - ASI - IFS - IEA - TPA |
| Research consultancy |  | - Comres - DWF - Ernst & Young - Euromonitor - Future Thinking - Investec - Liberum - Marketing Sciences - McKinsey Global Institute - Numis - Oxford Economics - Shore Capital - Simon-Kucher & Partners - Sucden Financial | - ComRes - DWF - EY - Euromonitor - Fut Think - Investec - Liberum - Mktg Sci - MGI - Numis - OxEc - Shore - S-K&P - Sucden Fin |
| Consumer group |  | - Centre for Science in the Public Interest - El Poder del Consumidor | - CSPI - EPC |
| Other |  | - Brighton Students Against Sugar Tax - St Mary's Catholic school | - BSAST - St Mary’s |

^(1)^ Colour indicates node colours used to highlight organisational type in the discourse network diagrams

^(2)^ Abbreviations as they appear in the network diagrams

**Table 2: Concept statements identified in the debate on the Soft Drinks Industry Levy**

| ***Problem definition*** |
| --- |
| Obesity/sugar consumption costs NHS/government/society |
| Population/country consumes too much sugar/SSBs |
| Population/country has a problem with obesity and diet related disease |
| Population/country has a problem with childhood obesity |
| SSB/sugar consumption is bad for public health |
| SSB/sugar consumption cause ill-health independently of obesity |
| SSBs are a particular problem for young people/those on low income |
| SSB/sugar consumption causes tooth decay |
| ***Problem driver*** |
| Consumer demand for sugar-free drinks is increasing |
| SSBs do not drive obesity |
| Industry puts profits before public health |
| Industry is already taking voluntarily action |
| Retailers promoting sugary products drive obesity |
| Industry/retailers can/do play a role in health promotion |
| ***Solution - strategy*** |
| Singling out sugar/SSBs is not the answer - comprehensive strategy is needed |
| Government action is required on obesity/sugar consumption |
| Government action is required on childhood obesity/sugar consumption |
| SSB/sugar tax are nanny state policies |
| Taxation is not the right approach |
| There are “better ways” of tackling obesity |
| Public supports action on high sugar food/drink products |
| SSB/sugar consumption / obesity should be controlled like alcohol and tobacco |
| Focus should be on solving inequalities |
| ***Solution – industry controls*** |
| Control of advertising/marketing of unhealthy food and drinks are needed |
| Control of promotions is needed |
| ***Solution – specifically reformulation*** |
| Reformulation should extend beyond SSBs/sugar |
| Reformulation, reducing sugar/portion size, is needed |
| ***Solution – individual responsibility*** |
| Individuals should be provided with information/education to improve food choices |
| Parents have a responsibility for children’s diets |
| ***Solution – SSB/sugar tax*** |
| Public supports taxing high sugar food/drink products |
| SSB/sugar tax unfair on consumers and will hit the poorest hardest |
| SSB tax will benefit health of the poorest most |
| SSB tax will result in switching |
| SSB/sugar tax will send a strong message/keep industry in line/prompt action |
| SSB/sugar tax is supported by evidence |
| SSB/sugar tax needed to/will reduce sugar consumption, by promoting reformulation |
| SSB/sugar tax needed to/will reduce sugar consumption, by reducing purchases |
| SSB/sugar tax needs to be high enough – the higher the tax the bigger the impact |
| SSB/sugar tax will damage the wider economy |
| SSB/sugar tax will/should improve population health |
| SSB/sugar tax will raise/save public funds |
| SSB/sugar tax would/might be illegal |
| ***Solution – SDIL*** |
| SDIL/SSB tax will damage the SDI and associated industries |
| SDIL/SSB tax is/would be useful as part of a package of measures |
| SDIL is flawed |
| SDIL too complex/costly to implement |
| SDIL/sugar tax unfair on SDI/disappointing |
